# Central and peripheral neuromuscular fatigue following ramp and rapid maximal voluntary isometric contractions

**DOI:** 10.3389/fphys.2024.1434473

**Published:** 2024-08-20

**Authors:** Benjamin Dalton, Garrett Hester, Michaela Alesi, Jacob McDougle, Michael Cooper, Trisha VanDusseldorp, Robert Buresh, Yuri Feito

**Affiliations:** ^1^ Wellstar College of Health and Human Services, Kennesaw State University, Kennesaw, GA, United States; ^2^ Bonafide Health, LLC, JDS Therapeutics, Harrison, NY, United States; ^3^ dNea Onnim Consultancy, StAugustine, FL, United States

**Keywords:** rate of torque development, contractile properties, transcranial magnetic stimulation, electromyogarphy, corticospinal, explosive

## Abstract

**Introduction:**

Maximal voluntary isometric contractions (MVICs) as a fatiguing modality have been widely studied, but little attention has been given to the influence of the rate of torque development. Given the established differences in motor command and neuromuscular activation between ramp and rapid MIVCs, it is likely performance fatigue differs as well as the underlying physiological mechanisms.

**Purpose:**

To compare responses for rapid and maximal torque following ramp and rapid MVICs, and the corresponding neuromuscular and corticospinal alterations.

**Methods:**

On separate visits, twelve healthy males (22.8 ± 2.5 years) performed fatiguing intermittent MVICs of the knee extensors with either 2 s (RAMP) or explosive (RAPID) ramp-ups until a 50% reduction in peak torque was achieved. Before and after each condition, maximal and rapid torque measures were determined from an MVIC. Additionally, peripheral (twitch parameters) and central (voluntary activation) fatigue, as well as rapid muscle activation, and cortical-evoked twitch and electromyographic responses were recorded.

**Results:**

Maximal and late-phase rapid torque measures (*p* ≤ 0.001; $\eta_{p\;}^{2}$ = 0.635–0.904) were reduced similarly, but early rapid torque capacity (0–50 ms) (*p* = 0.003; *d* = 1.11 vs. *p* = 0.054; *d* = 0.62) and rapid muscle activation (*p* = 0.008; *d* = 1.07 vs. *p* = 0.875; *d* = 0.06) decreased more after RAMP. Changes in peripheral fatigue, as indicated by singlet and doublet contractile parameters (*p* < 0.001 for all; $\eta_{p\;}^{2}$ = 0.752–0.859), and nerve-evoked voluntary activation (*p* < 0.001; $\eta_{p\;}^{2}=$ 0.660) were similar between conditions. Corticospinal inhibition (via silent period) was only increased after RAPID (*p* = 0.007; *d* = 0.94 vs. *p* = 0.753; *d* = 0.09), whereas corticospinal voluntary activation and excitability were unchanged.

**Conclusion:**

Ramp, fatiguing MVICs impaired early rapid torque capacity more than rapid MVICs, and this was accompanied by decrements in rapid muscle activation. Responses for peripheral and central fatigue (nerve and cortical stimulation) were largely similar between conditions, except that rapid MVICs increased corticospinal inhibition.

## 1 Introduction

Performance fatigue is commonly defined as the exercise-induced reduction in the ability of skeletal muscle to produce force or power (Barry and Enoka, 2007). Many physiological mechanisms can contribute to performance fatigue and, depending on the origin, these alterations may induce central or peripheral fatigue. Central fatigue, which is the reduction in voluntary activation of skeletal muscle during exercise, can result from spinal or supraspinal adjustments (Gandevia, 2001). Fatigue-related changes at or distal to the neuromuscular junction is considered peripheral fatigue and is indicated by a decrease in contractility (Place et al., 2010) or sarcolemmal excitability (Bigland-Ritchie et al., 1982) of skeletal muscle. Performance fatigue and the corresponding central and peripheral responses are dependent upon factors such as contraction type, muscle group, and contraction intensity (Enoka and Stuart, 1992; Linnamo et al., 1997; Babault et al., 2006; Hunter, 2018). Maximal voluntary isometric contractions (MVICs) are a common fatiguing modality (Taylor et al., 2000; Buckthorpe et al., 2014) for a few reasons. They induce fatigue within seconds, enable the identification of central and peripheral responses when used in conjunction with peripheral nerve or transcranial magnetic stimulation (TMS) (Bigland-Ritchie and Woods, 1984; Todd et al., 2003), and involve a consistent benchmark of “maximal” performance (Taylor and Gandevia, 2008). However, little attention has been given to the influence of the rate of torque (or force) development (RTD) on fatigue-induced performance or the corresponding neuromuscular changes.

Rapid (i.e., achieving peak torque (PT) as quickly as possible) and ramp (i.e., gradually increasing torque to achieve PT) MVICs involve differing neuromuscular activation strategies to achieve PT as the former emphasizes explosive torque production. Indeed, PT is similar for MVICs despite differences in instructions related to the speed of contraction (Sahaly et al., 2001; Sahaly et al., 2003; Jaafar and Lajili, 2018; Tomko et al., 2018). Rapid and ramp MVICs both involve maximal effort and control of other important factors (e.g., joint angle, contraction velocity), while allowing a comparison of distinctly different neuromuscular activation strategies for the production of torque (or force). Buckthorpe, Pain. Buckthorpe et al., 2014 examined the fatiguing effects of rapid MVICs, alone, on rapid torque capacity and PT and the former was more dramatically reduced which was a result of both central and peripheral mechanisms. It is plausible that fatiguing rapid and ramp MVICs affect rapid torque capacity and PT differently, and the accompanying neuromuscular alterations may be unique.

A fundamental difference between rapid and ramp MVICs is the rate at which the nervous system activates skeletal muscle. While the orderly recruitment of motor units is maintained (Henneman, 1957), recruitment of similar sized motor units occurs earlier (Büdingen and Freund, 1976; Desmedt and Godaux, 1977) and firing rates are substantially greater (>100 Hz vs. 20–50 Hz) during rapid contractions (Desmedt and Godaux, 1977; Heckman and Enoka, 2012a). It is possible the earlier recruitment of motor units, particularly more fatigable higher-threshold motor units, and the substantially higher firing rates associated with rapid MVICs exacerbates peripheral fatigue (e.g., metabolite accumulation and excitation-contraction coupling disturbance). Rapid and ramp MVICs also have prominent differences in their central nervous system control schemes, which could lead to unique corticospinal adjustments following fatiguing exercise. For example, it is believed that rapid movements are primarily preprogrammed such that there is little or no alterations to motor neuron activation via peripheral feedback (Desmedt and Godaux, 1979). Baudry and Duchateau (2021) found that increased excitability of the motor cortex preceded that of the spinal level during the preparatory phase of both rapid and ramp contractions. However, increased corticospinal excitability occurred later (closer to contraction onset) and more abruptly for rapid contractions, indicating the intended rate of torque development influences the timing and amplitude of corticospinal excitability. Further, at least for hand muscles, greater amplitudes for motor evoked potentials (MEPs) were shown at the onset (Kasai and Yahagi, 1999) and during skill acquisition (Muellbacher et al., 2001) of rapid but not ramp movements. Despite the fact the aforementioned evidence stems from motor control studies examining muscle contractions in a non-fatigued state, the expected differences in descending motor command and sensory-motor integration could be influential for the fatigue-induced corticospinal adjustments.

It is important to determine if the response for rapid and maximal torque capacity as well as the corresponding neuromuscular and corticospinal alterations are affected differently after fatiguing, rapid and ramp MVICs. Rapid torque capacity is critical for athletic performance (Tillin et al., 2013) and physical function in older adults (Osawa et al., 2018; Hester et al., 2020), so it is valuable to understand its response to fatigue. Insight on the neuromuscular underpinnings of fatigue following rapid contractions could guide future research in athletic or older populations where decrements have meaningful performance and physical function implications. A comprehensive approach incorporating both peripheral nerve and motor cortex stimulation is necessary to specifically identify the peripheral and central changes associated with these fatiguing conditions. The aim of this study was to compare responses for rapid and maximal torque following rapid and ramp MVICs, and the corresponding neuromuscular and corticospinal alterations. It was hypothesized that, given the same magnitude of fatigue (50% reduction in PT) between conditions, fatigue will occur more quickly, and rapid torque capacity will be reduced more after the rapid MVIC protocol. In addition, although the response for central fatigue is not expected to be different due to similar intensities (Carroll et al., 2017), peripheral fatigue will be greater after the rapid MVIC protocol.

## 2 Materials and methods

### 2.1 Participants

Twelve young (age: 22.8 ± 2.5 years, BMI: 26.3 ± 3.7 kg/m^2^, body fat %: 19.3% ± 7.0%), recreationally active, healthy males were recruited for this randomized, crossover design study. *A priori* analysis using G*Power (v. 3.0.10) software indicated a total sample size of 11 was needed to provide a statistical power of 0.80 with a medium effect size (*f* = 0.25). All subjects reported having performed 1–3 days per week of lower-body resistance training exercise and endurance exercise over the past 6 months. Individuals were excluded if they presented with any disease or illness, were not cleared for physical activity via the Physical Activity Readiness Questionnaire, had a BMI over 30, suffered a musculoskeletal injury of the lower-body within the past year, supplemented with creatine or beta-alanine, or reported running more than 60 min or six miles per week. Finally, any individual providing a “yes” response to any of the 13 questions on the TMS screening questionnaire (Rossi et al., 2011), using ear cochlear implants, presenting with any contraindications to wearing ear plugs, or currently take medication for depression or anxiety was excluded as well. Prior to data collection, this study was approved by the university Institutional Review Board under the Ethical Principles and Guidelines for the Protection of Human Subjects of Research report. All subjects provided oral and written consent prior to beginning the study.

### 2.2 Experimental design

**FIGURE 1:**
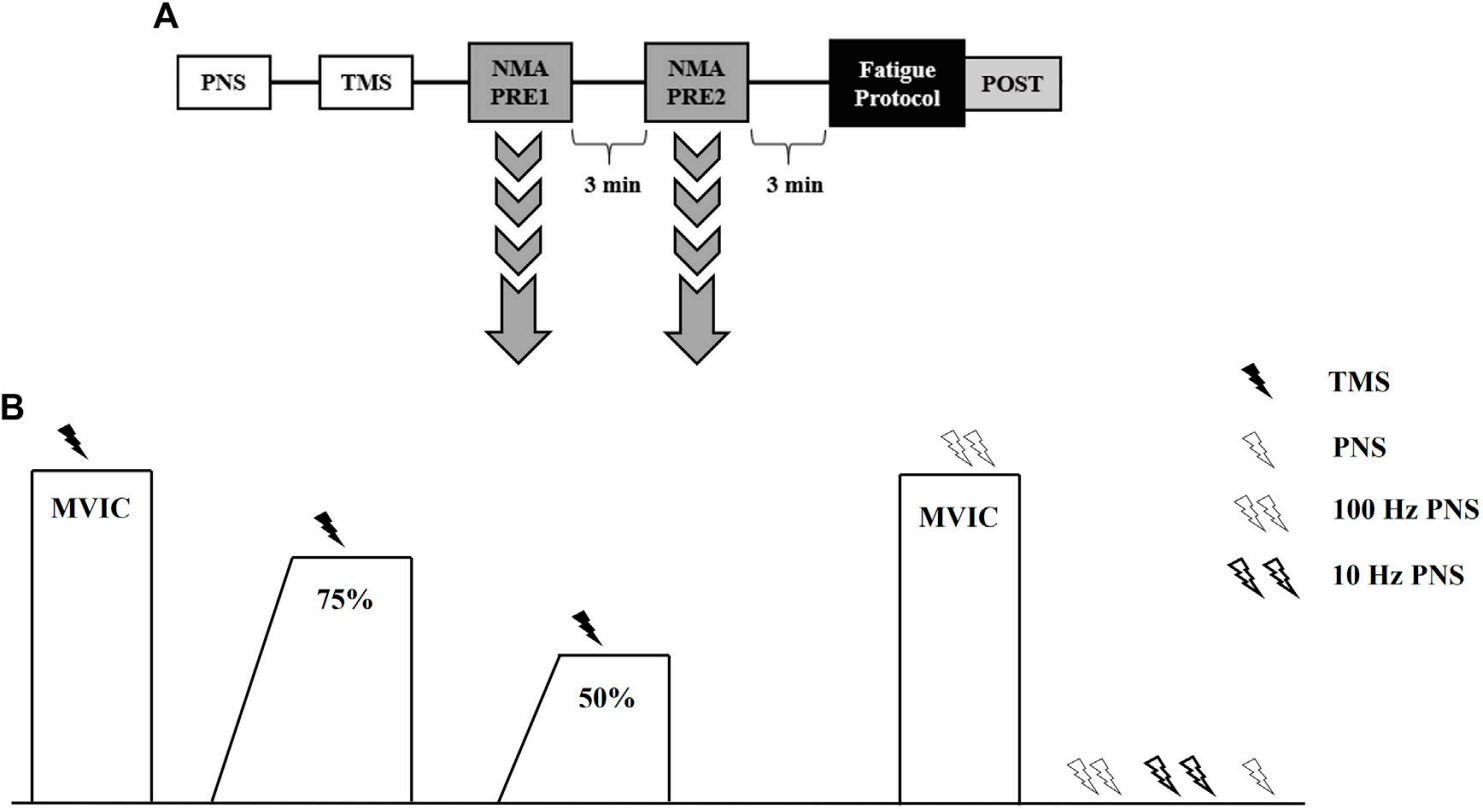
Timeline of procedures during testing visits **(A)**. Neuromuscular assessment (NMA) procedures used to assess central and peripheral fatigue responses **(B)**. TMS, transcranial magnetic stimulation; PNS, peripheral nerve stimulation; MVIC, maximal voluntary isometric contraction.

Participants completed three laboratory visits, separated by at least 4 days but not more than 7 days, at the same time (±2 h) of day. The first visit consisted of body composition testing and familiarization with testing procedures including TMS and PNS, as well as both fatiguing protocols. During the second and third visits, subjects completed fatiguing, intermittent MVICs consisting of either a 2 s torque ramp-up (RAMP) or explosive torque ramp-up (RAPID) in randomized order. Figure 1 depicts an overview of testing visits and the neuromuscular testing protocol (described below). Participants were instructed to complete a 4 h fast prior to the first visit only. In addition, subjects were instructed to avoid caffeine, alcohol, and vigorous exercise for 12, 24, and 48 h, respectively, before each visit. A 24-h dietary recall was performed prior to each testing visit to track carbohydrate, fat, and total caloric intake using MyFitnessPal (Version 22.10). No differences existed for carbohydrate (RAMP = 239 ± 78 g, RAPID = 196 ± 79 g; *p* = 0.119), fat (RAMP = 109 ± 77 g, RAPID = 75 ± 32 g; *p* = 0.204), or total kilocalories (RAMP = 2,404 ± 899 kcals, RAPID = 1834 ± 518 kcals; *p* = 0.082) intake between conditions.

### 2.3 Body composition

Height (cm) and body mass (kg) were measured using an electronic physician scale and stadiometer (Tanita WB 3000, Arlington Heights, IL, United States). Total body fat % was obtained via bioelectrical impedance analysis (InBody770, InBody Co., Cerritos, CA, United States) following manufacturer recommendations.

### 2.4 Torque and electromyography

Torque was recorded for the right knee extensors using a calibrated Biodex four isokinetic dynamometer (Biodex Medical Systems, Inc. Shirley, NY, United States), with knee and hip angles of 110° (full extension = 180°) and 100°, respectively. The lateral condyle of the femur was aligned with the axis of rotation of the dynamometer with the lower leg secured to the lever arm (∼2 cm superior to the medial malleolus). Participants were seated with hands across chest and restraining straps were placed over the trunk and pelvis. Electromyography (EMG) (Bagnoli Desktop System, Delsys, Inc. Natick, MA, United States) of the vastus lateralis was recorded using parallel bar, bipolar surface electrodes (DE-2.1, Delsys, Inc., Natick, MA, United States). A reference electrode was placed over the right patella. The skin was shaved, abraded, and cleaned with alcohol, and subsequently the electrodes were placed over the muscle belly in accordance with the recommendations of the SENIAM project (Hermens et al., 1999). Torque and EMG signals were sampled at 2 kHz using EMGworks software (Delsys, Inc., Natick, MA, United States).

### 2.5 Peripheral nerve stimulation

Percutaneous stimulation of the femoral nerve was performed using a constant-current stimulator (Digitimer DS7AH, Welwyn Garden City, United Kingdom). The cathode electrode (20 mm diameter; Technomed United States Inc., White Bear Township, MN) was taped on the skin over the femoral nerve in the femoral triangle and the anode electrode (40 × 50 mm diameter; Technomed United States Inc., White Bear Township, MN) was placed on the gluteal fold. The optimal location of the cathode was determined as the site that evoked the greatest twitch response from a single 100 μs pulse at a low current (80–100 mA). The stimulator current was then gradually increased by 10–30 mA increments until there was no further increase in either the twitch torque or M-wave of the vastus lateralis. Following this, the stimulator current was increased by 20% to ensure maximal activation and this current (RAMP: 384 ± 120 mA, RAPID: 387 ± 116 mA; *p* = 0.849) was used for all subsequent stimulations. A dolorimeter (Baseline; White Plains, NY) was used to aid in applying consistent pressure to the cathode during each stimulation.

### 2.6 Transcranial magnetic stimulation

A Magstim 200^2^ transcranial magnetic stimulator (Magstim Company Limited, Whitland, United Kingdom) was used to deliver single pulse stimuli over the left motor cortex via a concave double cone coil (110 mm diameter; Magstim Company Limited, Whitland, United Kingdom). Participants wore a cervical collar during testing to stabilize the head and neck. To determine the vastus lateralis hotspot, the vertex of the skull and twelve locations (1 cm increments along the sagittal and coronal planes) were marked on a wig cap. Stimuli at 50% (increased to 60% if there was no visible MEP or superimposed twitch) of maximal stimulator output were delivered at each location while subjects performed an isometric contraction of the knee extensors at 20% MVIC. A custom built laser attachment was used to assist with maintaining proper coil alignment throughout testing (Goss et al., 2012). The hotspot was determined as the location eliciting the greatest vastus lateralis MEP and minimal biceps femoris MEP and was used for each subsequent stimulation for that experimental session. Participants then performed four series of four isometric knee extensions at 20% MVIC during which the stimulator output was randomized between 50, 60, 70, and 80% of maximal stimulator output (10 s of rest between each contraction, ∼30 s rest between intensities) (Koral et al., 2020). The optimal intensity was determined as the lowest intensity that elicited the largest vastus lateralis MEP with a minimal biceps femoris MEP and this was used for all subsequent stimulations (Gruet et al., 2014). Despite conditions being randomized, optimal intensity was greater in the RAPID (64% ± 9%) condition compared to RAMP (60% ± 9%) (*p* = 0.007; *d* = 0.95).

### 2.7 Neuromuscular testing protocol

Peripheral nerve and TMS procedures, as described above, were performed similarly prior to neuromuscular testing. An overview of the neuromuscular testing protocol can be seen in Figure 1. Subjects performed two trials of the neuromuscular testing protocol, separated by 3 min, prior to fatigue (Koral et al., 2020). Another neuromuscular testing protocol was completed immediately (∼10 s) following the termination of the fatiguing protocol (Figure 1). Subjects began with a warm-up including two isometric knee extensions at 50% and 75% of perceived maximal effort. The neuromuscular testing protocol began with subjects performing an MVIC and TMS being delivered once they achieved their PT. Subjects were instructed to recontract as quickly as possible following TMS. Then, they relaxed for ∼10 s before following a template that displayed 75% of their PT from the previous MVIC. Once they achieved the 75% threshold, TMS was delivered similar to the preceding MVIC. Subjects then performed a 50% tracing and TMS was delivered in a similar manner. Subjects then rested for 5 s before performing another MVIC, during which doublet (100 Hz) nerve stimulation was delivered. The subject then remained relaxed while nerve stimuli were delivered in the order of a 100 Hz doublet, 10 Hz doublet, and a singlet. For all MVICs, subjects were instructed to “kick out as hard and fast as possible”. All submaximal isometric contractions utilized a template with 2 s ramp-up and a 5 s plateau at the relative torque amplitude. Strong verbal encouragement and visual biofeedback was provided during the testing and fatigue protocols.

### 2.8 Fatigue protocols

**FIGURE 2:**
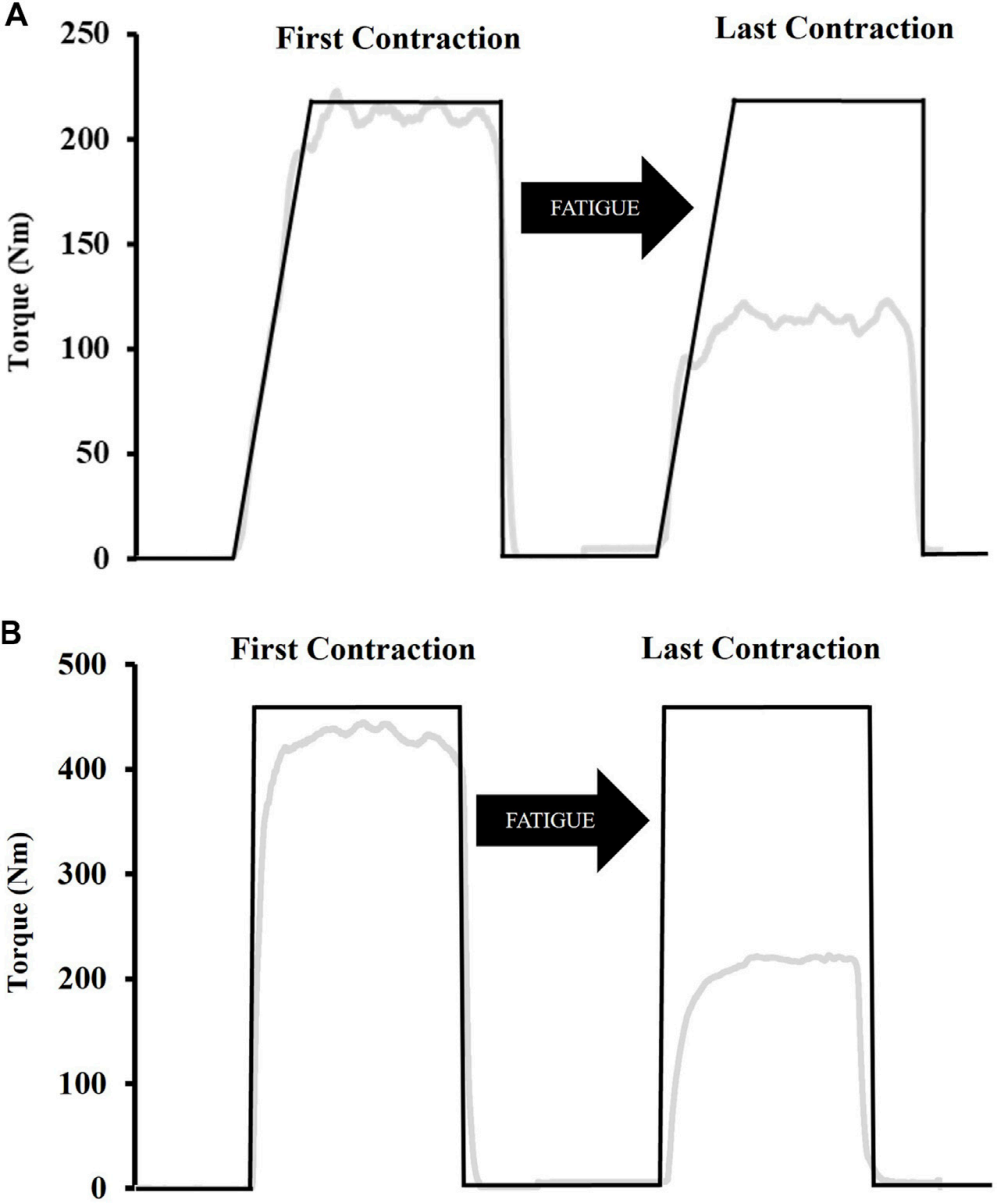
Examples of the template subjects followed during the fatiguing ramp **(A)** and rapid **(B)** protocols. The ramp protocol consisted of a 2 s ramp-up whereas participants were instructed to push “as fast and hard as possible” for the rapid protocol. Both protocols involved a 5 s “maximal effort” phase after which participants were instructed to immediately relax. The black line indicates the template while the grey line indicates torque in real-time.

Figure 2 shows an example of the RAMP and RAPID fatigue protocols. The two fatigue protocols consisted of intermittent MVICS with a 5 s “maximal effort” phase. The only difference between the two protocols was the RTD. The RAMP protocol consisted of a 2 s ramp-up prior to PT being achieved for each MVIC under the instruction of “push as hard as possible”. The RAPID protocol involved MVICs under the same instructions (“as fast and hard as possible”) as MVICs during testing. For each protocol, 5 s separated MVICs and the protocols were terminated once 50% of PT could not be achieved for two consecutive contractions. A template, which was based on the baseline PT, was displayed on a screen directly in front of the subjects. The template illustrated the corresponding slope for the fatigue protocol, and provided constant visual feedback of their target PT. The RAMP template demonstrated a 2 s ramp-up, whereas the RAPID template showed a straight line perpendicular to the resting baseline to emphasize rapid torque production. Rating of perceived exertion (RPE) (Category Ratio Scale-10) (Neely et al., 1992) was recorded immediately after the last contraction.

### 2.9 Data analysis

**FIGURE 3:**
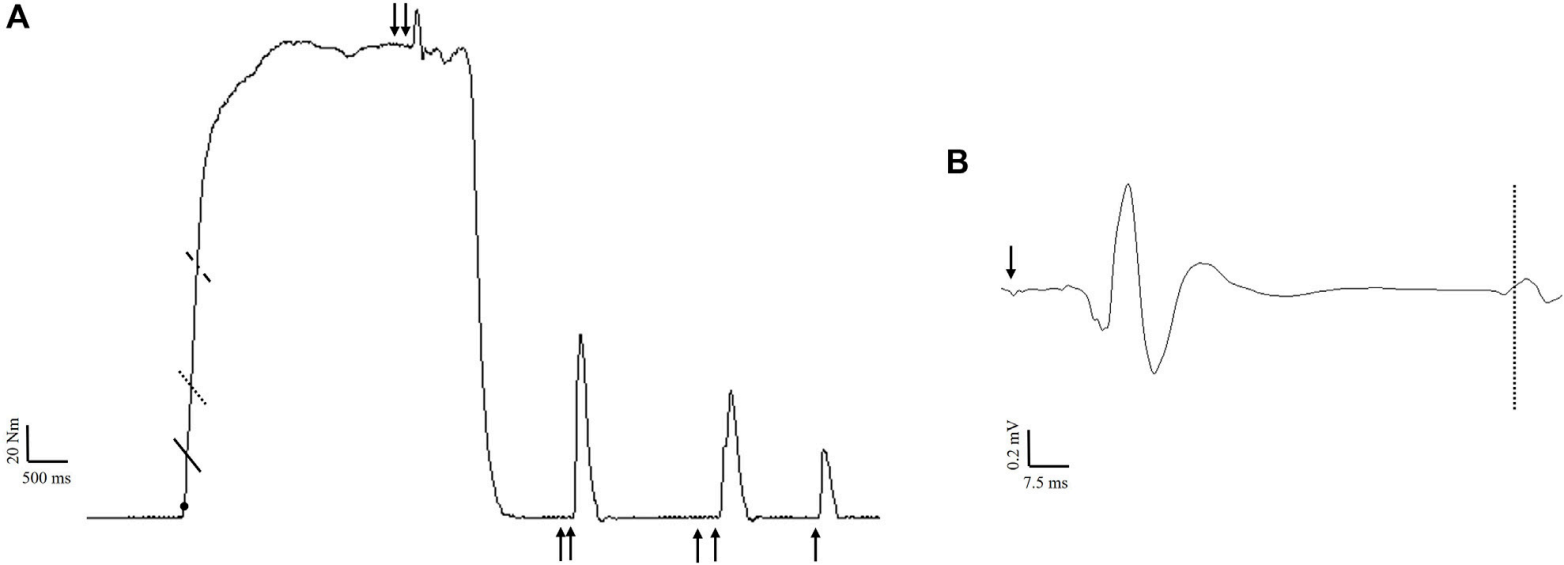
Example maximal voluntary isometric contraction **(A)** indicating the time intervals for rapid torque measures calculated from onset (filled circle) to 50 ms (solid line), 100 ms (dotted line), and 200 ms (dashed line). Nerve stimulation (100 Hz) was delivered during the contraction for the calculation of voluntary activation. During subsequent rest, 100 Hz, 10 Hz, and singlet nerve stimulation was performed to calculate contractile properties. Example of a motor evoked potential **(B)** following stimulation of the motor cortex (arrow). The dashed line indicates the resumption of electromyographic activity, which was used to calculate the corticospinal silent period.

The scaled, gravity corrected torque signal was digitally filtered with a zero lag, low-pass (150 Hz) Butterworth filter using custom written software (LabVIEW, National Instruments, Austin, TX). Impulse was calculated as the area under the torque-time curve (∫Torque d*t*) for both fatigue protocols. Isometric PT was considered the highest 500 ms rolling average during MVICs prior to any stimulation. Torque onset was set at 2.5 Nm (Hester et al., 2019), and RTD was obtained from the linear slope of the torque-time curve (Δtorque/Δtime) (Figure 3). Peak RTD (RTD_pk_) was considered the highest slope value from a 10 ms rolling average. RTD was also calculated from contraction onset to 50 (RTD_0-50_), 100 (RTD_0-100_), and 200 (RTD_0-200_) ms. Absolute torque (5 ms average) was determined at the same time intervals (TQ_50_, TQ_100_, TQ_200_, respectively). The zero means EMG signal was processed using a fourth order Butterworth filter with a low- and high-frequency cutoff of 10 and 500 Hz, respectively. EMG amplitude was calculated as the root mean square (RMS) for the 500 ms epoch corresponding to peak torque (250 ms either side) during MVICs. Fatigue-induced changes in maximal muscle activation were examined by normalizing EMG amplitude from each post-testing time point to the baseline value. As an indicator of rapid muscle activation, RMS during the first 50 ms (RMS_0-50_) was divided by EMG amplitude during the same contraction (Gerstner et al., 2017). RMS_0-50_ was also normalized to m-wave amplitude to better account for changes in sarcolemmal excitability (Bigland-Ritchie et al., 1982).

Voluntary activation was calculated from femoral nerve (VA_FNS_) and cortical (VA_TMS_) stimulation as (1 – superimposed twitch/resting twitch) × 100 (Merton, 1954; Gandevia, 2001). For VA_FNS_, a correction factor was applied as described by Strojnik and Komi (Strojnik and Komi, 1998) since stimulation was not always applied during peak torque (Figure 3). For VA_TMS_, the resting twitch was estimated using linear regression between voluntary torque and superimposed torque derived from the isometric contractions performed at 50%, 75%, and 100% intensity (Goodall et al., 2009). A linear relationship was verified before (*r* ^2^ = 0.93 ± 0.07 and after (*r* ^2^ = 0.90 ± 0.10) fatigue. Due to a non-linear relationship between responses, one subject had to be excluded from VA_TMS_ analysis (n = 11). M-wave peak-to-peak amplitude was derived from the singlet delivered at rest. To assess corticospinal excitability, MEP peak-to-peak amplitude from each testing intensity was normalized to the M-wave peak-to-peak at the corresponding timepoint (Hunter et al., 2006). Corticospinal inhibition was assessed via the silent period, which was determined visually and defined as the time from MEP onset to the return of continuous EMG (Damron et al., 2008) (Figure 3). Since increasing the number of trials increases reliability for MEP measurements (Goldsworthy et al., 2016) and contraction intensity was not of interest, the average (across contraction intensities) normalized MEP amplitude and silent period was used for statistical analysis. The ratio of the resting peak twitch amplitudes produced by 10:100 Hz doublets was used as an index of low-frequency fatigue (Edwards et al., 1977; Verges et al., 2009) (Figure 3). Contractile properties including peak twitch, normalized time to peak twitch, twitch RTD, and half relaxation time were calculated from the singlet.

### 2.10 Statistical analyses

Data normality was assessed through assessment of skewness and kurtosis. Specifically, skewness and kurtosis values were divided by their standard error and a threshold of 1.96 was used to determine non-normality (Field, 2009). All dependent variables were normally distributed, except for peak twitch, twitch RTD, and RMS_0-50_. Dependent samples t-tests were used to compare baseline values between conditions. Two-way repeated measures of analyses of variance [condition (RAMP vs. RAPID) × time (PRE vs. POST)] were used to analyze changes in all normally distributed dependent variables. Upon a significant two-way interaction, Bonferroni-corrected pairwise comparisons on the simple effects were performed. Mauchley’s test was used to assess sphericity, and a Greenhouse-Geisser correction factor was applied when the assumption of sphericity failed. For non-normally distributed variables, a Wilcoxon Signed Ranks Test was performed within each condition. Statistical analyses were performed with SPSS version 28 (IBM Corporation, Chicago, IL). An alpha level of *p* ≤ 0.05 was used to indicate statistical significance. Effect sizes were reported using partial eta squared ( $\eta_{p}^{2}$ ) for ANOVA analyses and <0.06, 0.07–0.14, and >0.14 indicated small, medium, and large effect sizes, whereas Cohen’s *d* was used for pairwise comparisons with 0.20, 0.50, and 0.80 indicating the same effect size (Cohen, 1988). Data are reported as mean ± SD in tables and text, and mean and individual data are provided in figures.

## 3 Results

### 3.1 Baseline visits and fatiguing protocols

There were no differences in any muscle performance or neuromuscular outcomes for the baseline visits (*p* = 0.141–0.968; *d* = 0.01–0.45). As expected, percent decline during RAMP (−49.93% ± 4.17%) and RAPID (−50.06% ± 3.45%) were similar (*p* = 0.923; *d* = 0.029). The average RTD_0-200_ for RAMP and RAPID was 182.21 ± 54.25 Nm·s^-1^ and 585.13 ± 149.33 Nm·s^-1^, respectively (*p* < 0.001; d = 2.75). Impulse was similar between fatigue protocols (RAMP = 26,873 ± 7,774.44 Nm·s, RAPID = 26,045 ± 6,004 Nm·s; *p* = 0.604; *d* = 0.15), but the number of MVICs performed was greater for RAPID (RAMP = 22 ± 9, RAPID = 26 ± 7; *p* = 0.017; *d* = 0.80). RPE was similar after both conditions (RAMP = 7.58 ± 1.83, RAPID = 7.50 ± 1.38; *p* = 0.820; *d* = 0.067)).

### 3.2 Voluntary isometric PT and rapid torque measures

**FIGURE 4:**
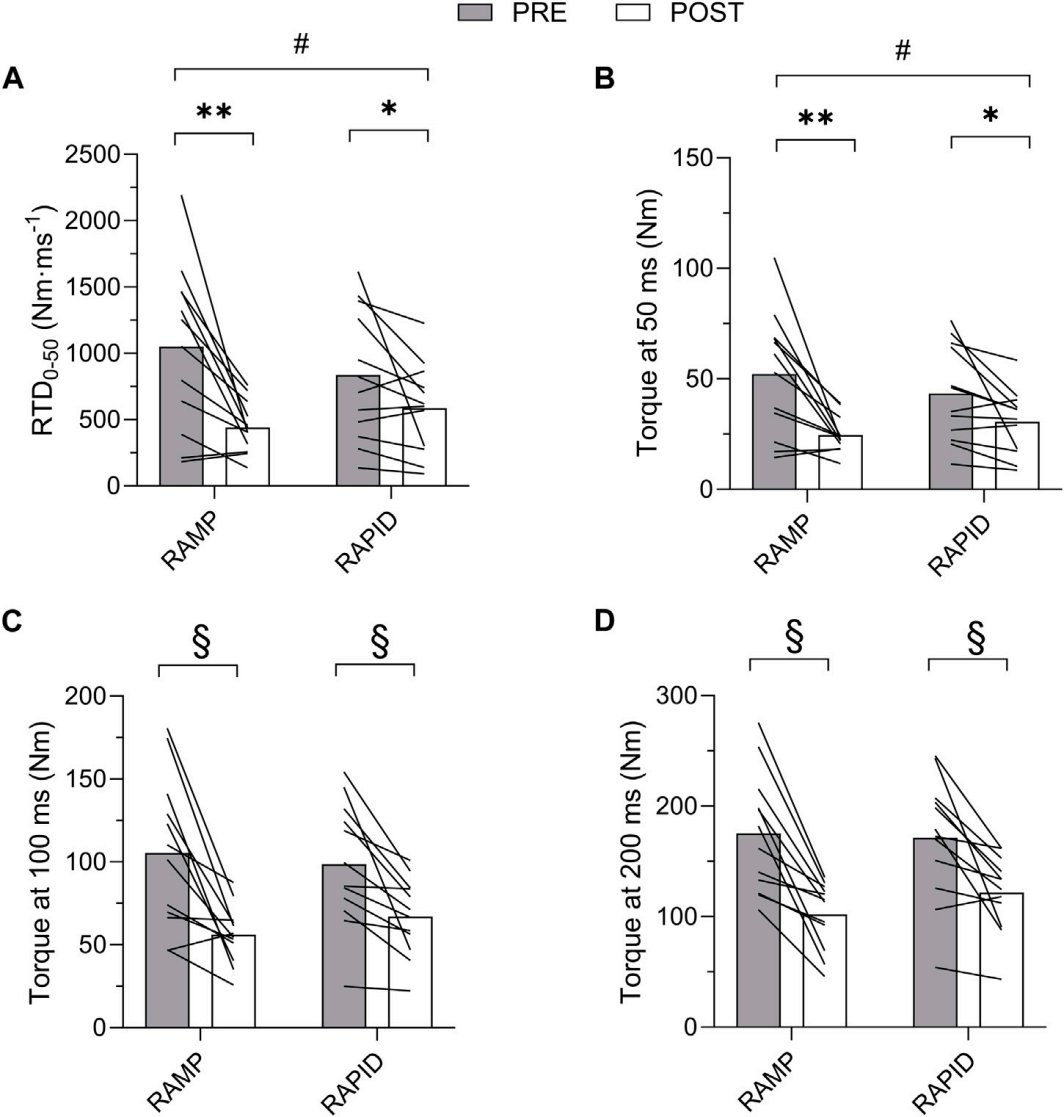
Rate of torque development (RTD) from 0 to 50 ms **(A)** and absolute torque at 50 **(B)**, 100 **(C)**, and 200 **(D)** ms before and after each fatiguing condition. # indicates two-way interaction. ** indicates greater decrease following RAMP compared to RAPID. * indicates decrease compared to pre. § indicates similar decrease compared to pre between conditions.

Data for PT and all rapid torque measures as well as two-way ANOVA findings are shown in Table 1. Means and individual level data for RTD_0-50_, TQ_50_, TQ_100_, and TQ_200_ are shown in Figure 4. A two-way interaction was only found for TQ_50_ and RTD_0-50_, whereas all other rapid torque measures and PT demonstrated a main effect for time indicating similar reductions between conditions. Pairwise comparisons revealed greater reductions for TQ_50_ after RAMP (*p* = 0.003; *d* = 1.09) compared to RAPID (*p* = 0.028; *d* = 0.73). Similarly, RTD_0-50_ decreased more after RAMP (*p* = 0.003; *d* = 1.11) compared to RAPID (*p* = 0.054; *d* = 0.62) (Table 1; Figure 4).

**TABLE 1:** Effects of RAMP and RAPID on isometric PT and rapid torque measures.

Variable	RAMP		RAPID		Two-way interaction	Time effect
	PRE	POST	PRE	POST		
PT (Nm)	315.44 ± 56.76	217.59 ± 31.21	319.07 ± 61.39	214.12 ± 30.54	*p* = 0.388; $\eta_{p\;}^{2}=$ 0.068	***p* < 0.001;** $\eta_{p\;}^{2}=$ **0.904**
TQ_50_ (Nm)	52.16 ± 27.75	24.61 ± 8.16	43.35 ± 21.85	30.52 ± 14.55	***p* = 0.031;** $\eta_{p\;}^{2}=$ **0.357**	***p* = 0.004;** $\eta_{p\;}^{2}=$ **0.548**
TQ_100_ (Nm)	105.36 ± 46.01	56.13 ± 17.41	98.51 ± 37.69	67.07 ± 23.22	*p* = 0.111; $\eta_{p\;}^{2}=$ 0.214	***p* = 0.001;** $\eta_{p\;}^{2}=$ **0.638**
TQ_200_ (Nm)	175.44 ± 54.63	102.01 ± 30.22	171.49 ± 55.86	121.90 ± 34.66	*p* = 0.156; $\eta_{p\;}^{2}=$ 0.174	***p* < 0.001;** $\eta_{p\;}^{2}=$ **0.738**
RTD_pk_ (Nm·s^-1^)	1,605.56 ± 653.03	963.43 ± 243.93	1,545.57 ± 557.66	1,092.54 ± 347.54	*p* = 0.130; $\eta_{p\;}^{2}=$ 0.196	***p* = 0.001;** $\eta_{p\;}^{2}=$ **0.635**
RTD_0-50_ (Nm·s^-1^)	1,049.26 ± 619.39	441.40 ± 192.39	835.72 ± 495.16	587.41 ± 338.91	***p* = 0.012;** $\eta_{p\;}^{2}=$ **0.453**	***p* = 0.005;** $\eta_{p\;}^{2}=$ **0.520**
RTD_0-100_ (Nm·s^-1^)	1,151.74 ± 532.39	594.93 ± 202.64	1,054.77 ± 428.90	721.65 ± 277.44	*p* = 0.070; $\eta_{p\;}^{2}=$ 0.267	***p* = 0.001;** $\eta_{p\;}^{2}=$ **0.635**
RTD_0-200_ (Nm·s^-1^)	902.98 ± 281.19	544.88 ± 176.58	910.96 ± 307.84	651.01 ± 183.31	*p* = 0.263; $\eta_{p\;}^{2}=$ 0.112	***p* < 0.001;** $\eta_{p\;}^{2}=$ **0.718**

PT, peak torque; TQ_50_, torque at 50 ms; TQ_100_, torque at 100 ms; TQ_200_, torque at 200 ms; RTD_PK_, peak rate of torque development; RTD_0-50_, rate of torque development 0–50 ms; RTD_0-100_, rate of torque development 0–100 ms; RTD_0-200_, rate of torque development 0–200 ms

### 3.3 Contractile properties

**FIGURE 5:**
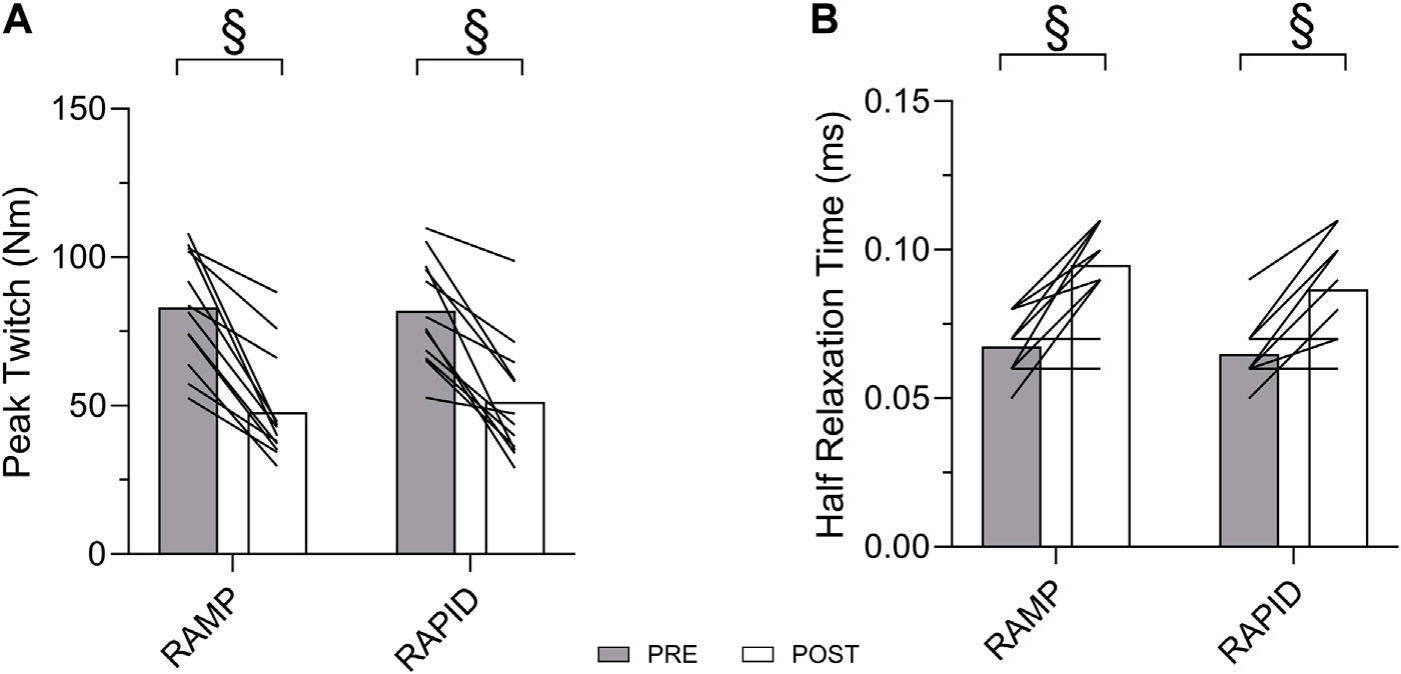
Peak twitch **(A)** and half relaxation time **(B)** before and after each fatiguing condition. § indicates similar change compared to pre between conditions.

Data for all contractile properties and two-way ANOVA findings are provided in Table 2. Means and individual level data for peak twitch and half relaxation time are shown in Figure 5. All contractile properties exhibited a main effect for time indicating a decrease at post, but no two-way interactions were found. Peak twitch and twitch RTD demonstrated similar reductions after RAMP (Wilcoxon Test; *p* = 0.002; *d* = 2.05 and Wilcoxon Test; *p* = 0.002; *d* = 1.83, respectively) and RAPID (Wilcoxon Test; *p* = 0.002; *d* = 1.83 and Wilcoxon Test; *p* = 0.003; *d* = 1.62, respectively). A two-way ANOVA showed the same result for these outcomes.

**TABLE 2:** Effects of RAMP and RAPID on contractile properties.

Variable	RAMP		RAPID		Two-way interaction	Time effect
	PRE	POST	PRE	POST		
Peak twitch (Nm)	83.08 ± 19.14	47.86 ± 18.43	81.98 ± 17.82	51.32 ± 20.07	*p* = 0.081; $\eta_{p\;}^{2}=$ 0.251	***p* < 0.001;** $\eta_{p\;}^{2}=$ **0.814**
Time to peak twitch (ms·Nm^-1^)	1.09 ± 0.20	1.74 ± 0.42	1.11 ± 0.21	1.82 ± 0.62	*p* = 0.686; $\eta_{p\;}^{2}=$ 0.015	***p* < 0.001;** $\eta_{p\;}^{2}=$ **0.752**
HRT (ms)	0.066 ± .0087	0.095 ± .015	0.065 ± .010	0.085 ± .016	*p* = 0.062; $\eta_{p\;}^{2}=$ 0.281	***p* < 0.001;** $\eta_{p\;}^{2}=$ **0.777**
Twitch RTD (Nm·s^-1^)	2,222.31 ± 488.04	1,371.89 ± 471.04	2,224.62 ± 450.52	1,428.81 ± 464.87	*p* = 0.552; $\eta_{p\;}^{2}=$ 0.033	***p* < 0.001;** $\eta_{p\;}^{2}=$ **0.785**
100 Hz peak twitch (Nm)	122.27 ± 29.93	85.87 ± 31.28	124.63 ± 27.22	88.83 ± 35.98	*p* = 0.882; $\eta_{p\;}^{2}=$ 0.002	***p* < 0.001;** $\eta_{p\;}^{2}=$ **0.782**
10 Hz peak twitch (Nm)	127.14 ± 33.83	73.30 ± 34.27	127.29 ± 30.74	78.01 ± 36.93	*p* = 0.442; $\eta_{p\;}^{2}=$ 0.055	***p* < 0.001;** $\eta_{p\;}^{2}=$ **0.859**
10 Hz:100 Hz peak twitch	1.03 ± 0.075	0.84 ± 0.136	1.00 ± 0.086	0.087 ± 0.126	*p* = 0.262; $\eta_{p\;}^{2}=$ 0.113	***p* < 0.001;** $\eta_{p\;}^{2}=$ **0.786**

HRT, half relaxation time; RTD, rate of torque development

### 3.4 Muscle activation

**FIGURE 6:**
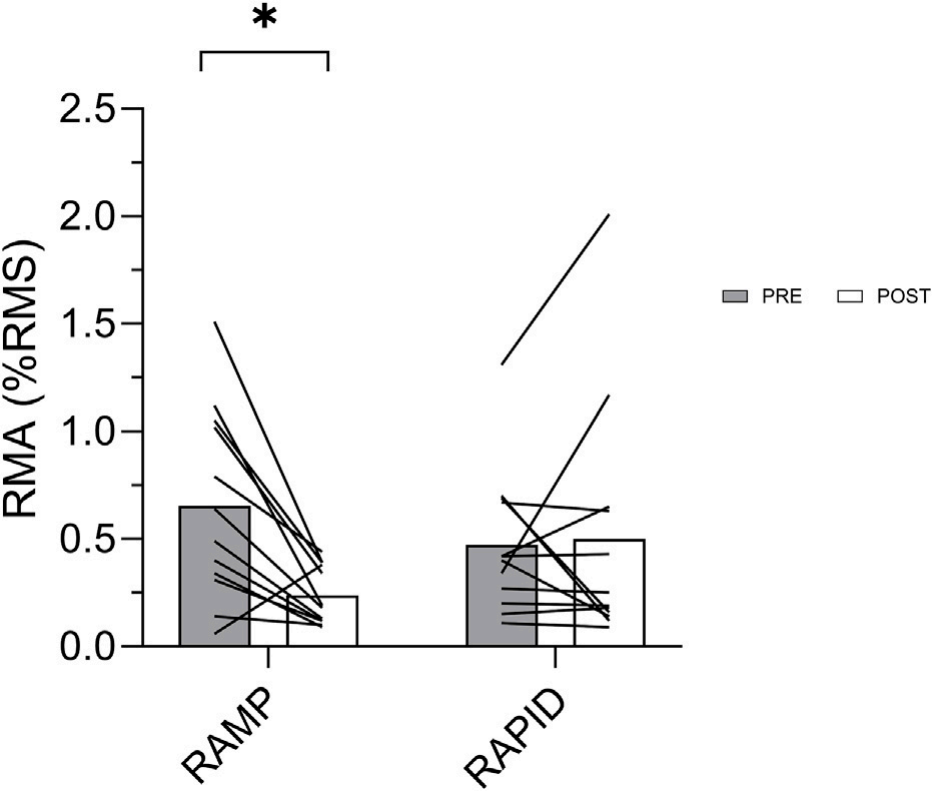
Rapid muscle activation (RMA) before and after each fatiguing condition. * indicates decrease compared to pre.

Maximal muscle activation was similar (*p* = 0.188; *d* = 0.405) following RAMP (1.06 ± 0.27 %RMS) and RAPID (0.95 ± 0.27 %RMS). RMS_0-50_, relative to maximal EMG amplitude, was reduced following RAMP (Wilcoxon Test; *p* = 0.008; *d* = 1.07; pre = 0.65 ± 0.44 %RMS, post = 0.23 ± 0.13 %RMS) but not RAPID (Wilcoxon Test; *p* = 0.875; *d* = 0.06; pre = 0.47 ± 0.33 %RMS, post = 0.50 ± 0.57 %RMS) (Figure 6). The same result was also found via two-way ANOVA interaction and subsequent pairwise comparisons. Similarly, RMS_0-50_ normalized to m-wave amplitude, was reduced following RAMP (Wilcoxon Test; *p* = 0.015; *d* = 0.74; pre = 0.044 ± 0.036%, post = 0.184 ± 0.009%) but not RAPID (Wilcoxon Test; *p* = 0.754; *d* = 0.15; pre = 0.034 ± 0.021%, post = 0.029 ± 0.026%).

### 3.5 Voluntary activation and corticospinal responses

**FIGURE 7:**
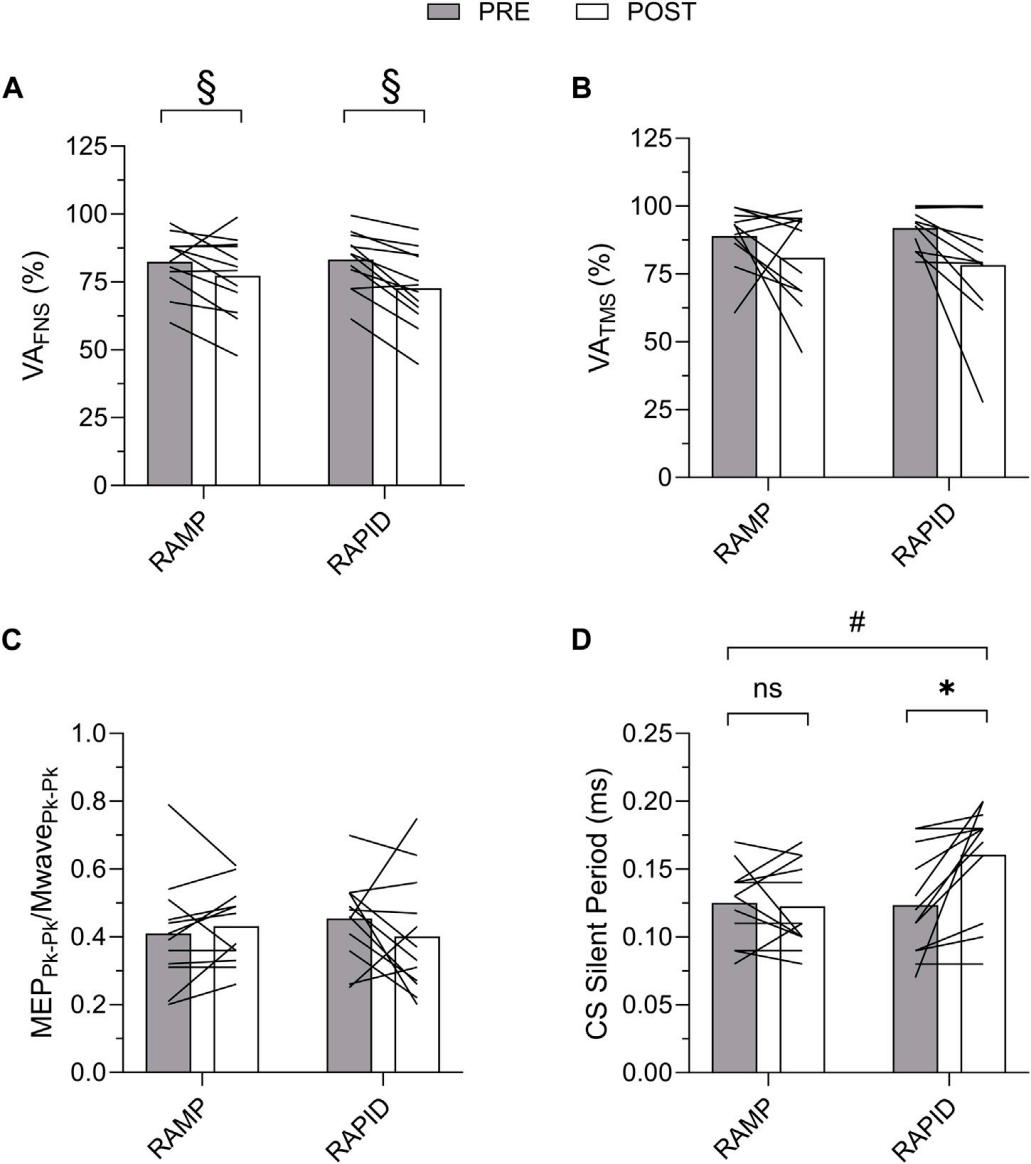
Voluntary activation with femoral nerve stimulation (VA_FNS_) **(A)** and transcranial magnetic stimulation (VA_TMS_) **(B)**, and corticospinal excitability **(C)** and corticospinal inhibition **(D)** before and after each fatiguing condition. MEP, motor evoked potential; CS, corticospinal; ns, non-significant. # indicates two-way interaction. * indicates increase only after RAPID. § indicates similar decrease compared to pre between conditions.

Voluntary activation as well as corticospinal excitability and inhibition data along with the two-way ANOVA findings are provided in Table 3. The means and individual level data are also shown in Figure 7. There was no two-way interaction for VA_FNS_, but a main effect of time was found indicating a similar reduction following both conditions. VA_TMS_ remained unchanged, regardless of condition, despite a relatively large effect size. There was no two-way interaction nor main effect for time for corticospinal excitability. Corticospinal inhibition demonstrated a two-way interaction. Pairwise comparisons determined it was increased (longer silent period) following RAPID (*p* = 0.007; *d* = 0.94) and remained unchanged after RAMP (*p* = 0.753; *d* = 0.09).

**TABLE 3:** Effects of RAMP and RAPID on voluntary activation and corticospinal responses.

Variable	RAMP		RAPID		Two-way interaction	Time effect
	PRE	POST	PRE	POST		
VA_FNS_ (%)	82.40 ± 10.56	77.20 ± 14.45	83.31 ± 10.60	72.62 ± 14.09	*p* = 0.118; $\eta_{p\;}^{2}=$ 0.207	***p* < 0.001;** $\eta_{p\;}^{2}=$ **0.660**
VA_TMS_ (%)	88.99 ± 11.33	81.02 ± 17.52	91.94 ± 7.34	78.26 ± 21.23	*p* = 0.971; $\eta_{p\;}^{2}<$ 0.000	*p* = 0.061; $\eta_{p\;}^{2}=$ 0.308
CS excitability (MEP_Pk-Pk_/Mwave_Pk-Pk_)	0.41 ± 0.16	0.43 ± 0.11	0.45 ± 0.12	0.40 ± 0.17	*p* = 0.166; $\eta_{p\;}^{2}=$ 0.167	*p* = 0.599; $\eta_{p\;}^{2}=$ 0.026
CS silent period (ms)	123.90 ± 26.01	121.39 ± 30.85	125.02 ± 38.38	161.36 ± 40.87	***p* = 0.013;** $\eta_{p\;}^{2}=$ **0.444**	***p* = 0.034;** $\eta_{p\;}^{2}=$ **0.346**

VA_FNS_, voluntary activation with peripheral nerve stimulation; VA_TMS_, voluntary activation with transcranial magnetic stimulation; CS, corticospinal; MEP, motor-evoked potential.

## 4 Discussion

The aim of this study was to compare responses for rapid and maximal torque following rapid and ramp MVICs, and the corresponding neuromuscular and corticospinal alterations. The RTD during the RAMP (2 s) and RAPID (as fast as possible) fatiguing protocols was closely aligned, albeit isometric in nature, with training paradigms focused on performing “heavy” and “explosive” resistance exercises, respectively. The current study was concerned with examining fatigue-related responses to these unique neuromuscular activation strategies, while controlling for differences in contraction type, velocity, and intensity. It was determined that fatigue-related changes in rapid torque capacity and some neuromuscular alterations were dependent upon condition. In contrast with our hypothesis, it was determined that early rapid torque capacity (0–50 m) was reduced more after RAMP. Reductions in peripheral fatigue and voluntary activation, as indicated by nerve stimulation, were similar between conditions. Despite no fatigue-related decrements for corticospinal drive after either condition, divergent changes were found for rapid muscle activation and corticospinal inhibition (silent period).

We hypothesized that, given the similar degree of fatigue (50% reduction in PT), RAPID would reach this threshold with fewer contractions due to greater peripheral fatigue, but this was not the case. The relatively closely aligned MVIC durations were selected to minimize the effect of contraction time to ensure the primary difference between conditions was the initial neuromuscular activation strategy. Our results indicated that isometric exercise with even a modestly longer contraction time (∼0.25–0.50 s), despite having a similar duration of maximal effort, induces fatigue more quickly than isometric exercise with an initial rapid intent. Despite fewer MVICs being needed to reach the 50% threshold for RAMP, the same impulse was achieved between conditions, so fatigue-induced differences between conditions were not influenced by differences in time under tension. In contrast to our hypothesis, early rapid torque capacity (0–50 ms) was reduced more following RAMP whereas all other torque measures were decreased similarly between conditions. Previous research has shown that rapid torque measures exhibit more dramatic decrements than PT following fatiguing exercise of the knee extensors (Minshull and James, 2013; Buckthorpe et al., 2014; Battazza et al., 2019). Buckthorpe et al. (2014) demonstrated this after rapid MVICs, but no comparisons were made with other contraction types. Based on our findings, slower, ramp MVICs have a more detrimental effect on early rapid torque capacity in particular, but this was not due to greater peripheral fatigue. We believed the earlier recruitment and correspondingly higher firing rates (Büdingen and Freund, 1976; Desmedt and Godaux, 1977; Heckman and Enoka, 2012b), particularly of higher-threshold glycolytic MUs during RAPID, would result in a greater metabolite accumulation and thus peripheral fatigue. In contrast, all indices of peripheral fatigue were affected similarly between conditions suggesting that metabolite accumulation and any impairments in cross-bridge cycling (Fitts, 1994) were not different. It is also reasonable to believe that the slower ramp-up (i.e., longer individual MVICs) associated with RAMP may induce greater peripheral fatigue as a result of greater blood flow occlusion (Russ and Kent-Braun, 2003), but this was not the case. The ramp-up duration was likely too short to have dramatic occlusive effects.

A larger decrement in rapid muscle activation was found after RAMP, which was likely influential for corresponding decreases in early rapid torque capacity. RMS_0-50_, whether normalized to maximal EMG or m-wave amplitude, showed a similar result suggesting that peripheral mechanisms such as impaired sarcolemmal transmission were not influential. Rapid torque capacity, particularly the early phase, derives a large contribution from initial neural drive (Klass et al., 2008; Folland et al., 2014; Del Vecchio et al., 2019). Rapid muscle activation, though indirect, reflects initial motor recruitment and firings (Van Cutsem et al., 1998; Klass et al., 2008), thus, slowing of MU behavior during the initial phase of the MVICs likely contributed to the attenuated rapid torque capacity. Given our methodological approach, the mechanism responsible for potential changes in MU behavior is unclear. While central fatigue would appear to not be responsible, since it was not affected more after RAMP, it is oversimplistic to rely on findings derived from superimposed stimulation during the plateau phase of MVICs to explain neuromuscular function during the early phase of a rapid MVICs. Additionally, more recently, the reticulospinal tract has been implicated as having a particularly prominent role in facilitating neural drive during rapid contractions (Škarabot et al., 2022). Responses for the reticulospinal tract were not assessed in the present study, but future research should consider assessment of this tract to possibly differentiate corticospinal and reticulospinal adjustments following fatigue.

In line with our hypothesis, there were no differences between conditions for central fatigue, but unique corticospinal adjustments were exhibited. Central fatigue (VA_FNS_), as indicated by nerve stimulation, was similar following both conditions which is likely because both involved intermittent contractions, the same intensity (maximal effort), and consequently were relatively short in duration (<5 min). Voluntary activation was also calculated based on stimulation of the motor cortex (VA_TMS_) to determine if supraspinal changes were influential for diminished corticospinal drive. Surprisingly, VA_TMS_ did not demonstrate a statistically significant reduction following either protocol, though a large effect size was indicated for the time effect. Typically, longer duration or sustained isometric fatigue protocols reduce central fatigue more dramatically compared to intermittent MVICs (Behm and St-Pierre, 1997; Bilodeau, 2006; Carroll et al., 2017). For example, Cairns et al. (2017) demonstrated that roughly 50% of subjects did not express central fatigue following a fatiguing, intermittent MVIC protocol similar to the present protocol. Additionally, Bilodeau et al. (2006) found central fatigue to be delayed during intermittent MVICs compared to a sustained MVIC task, and it was restored within 5 s of recovery. Though comparisons are difficult due to differing testing methodologies, VA_TMS_ was likely not prominent in the present study due its modest sensitivity to a protocol consisting of intermittent MVICs and because it was only measured after (not during) exercise. Similarly, corticospinal excitability remained unchanged following both conditions, which taken together, suggest that, despite central fatigue being present (reduced VA_FNS_), supraspinal excitability was not reduced and as a result net corticospinal drive was not impaired. There are consistent reports for a fatigue-induced reductions in VA_TMS_ and corticospinal excitability following *sustained* isometric contractions of the upper-and lower-body (Kennedy et al., 2016; Senefeld et al., 2018; Koral et al., 2020). However, similar to the present study, several studies have found no change in corticospinal excitability following *intermittent* isometric exercise of the knee extensors (Hilty et al., 2011; Gruet et al., 2014; Ansdell et al., 2019). Increased corticospinal inhibition was found after RAPID but not RAMP, and the reason for the latter is unclear as increased corticospinal inhibition is typical following intermittent MVICs (Taylor et al., 2000; Mileva et al., 2012). Some caution is needed for interpreting this finding, despite conditions being randomized and pre-testing procedures being similar, the stimulation intensity was higher for the RAPID condition. Stimulation intensity increases the silent period (Wilson et al., 1993; Säisänen et al., 2008), so this could have influenced this finding although it is unclear why baseline differences between conditions were not exhibited. Taylor et al. (2000) elegantly determined that central fatigue responses were similar following intermittent elbow flexion MVICs of various duty cycles. It is noteworthy that the smallest increase in corticospinal inhibition was found for the lowest 50% duty cycle (i.e., 5 s contract and 5 s relax), which is the same as the current protocol. Group III/IV afferent feedback is a strong mediator of corticospinal inhibition (Hilty et al., 2011), so the greater recovery time between MVICs allotted with a 50% duty cycle could also help explain the attenuated inhibitory response previously reported and our finding of no change after RAMP. Peripheral fatigue was similar between conditions, so it is unlikely feedback from metabosensitive receptors was influential for the discrepancy in corticospinal inhibition between conditions. Additional research is needed to verify our finding of greater corticospinal inhibition following rapid MVICs, and if the origin is specific to intracortical or spinal circuitry (Inghilleri et al., 1993; Ziemann et al., 1993).

The present study has several limitations. Our findings are limited to fatigue immediately following our conditions as no information was ascertained during fatiguing exercise or the minutes following during recovery. There were concerns that the number of muscle contractions required for the neuromuscular testing protocol would not provide sufficient recovery time between recovery testing intervals. We opted not to perform the number of stimulations necessary to gain neuromuscular insight *during* the fatiguing protocols. Subjects were well informed and familiarized on the differences in the ramp-up portion of the RAMP and RAPID conditions. However, despite our efforts and the use of a tracing template, adherence to the 2 s ramp-up template was inherently variable so the RTD was not precise for each MVIC. Nonetheless, while a substantially slower RTD for RAMP (e.g., 10 s) would have yielded a more divergent comparison, we felt it was important to focus on mimicking RTD expressed in “heavy” and “explosive” resistance exercise. Our findings are limited to rapid MVICs, and despite their prevalence in the literature, it would be worthwhile for future research to examine the influence of RTD during submaximal fatiguing contractions.

## 5 Conclusion

The present study determined that the RTD during fatiguing MVICs influences the subsequent performance decrements and physiological adjustments. We compared MVICs with a 2 s ramp-up and an explosive ramp-up (i.e., as fast as possible) to determine the effects of the different neuromuscular activation strategies on fatigue while controlling for other factors such as contraction type, velocity, and intensity. Despite a similar fatigue-induced reduction in peak strength (50%), ramp MVICs impaired early rapid torque capacity more than rapid MVICs, and this was accompanied by decrements in rapid muscle activation. Responses for peripheral and central fatigue were similar between conditions, except that only rapid MVICs increased corticospinal inhibition. Even when modest, a longer contraction duration during ramp MVICs appears to be more influential, compared to an “explosive” MVIC, for impairing performance and this decrement is exhibited primarily through attenuated rapid torque capacity during the early phase of contraction. The exact physiological underpinnings are not clear, but factors implicated in the impaired ability to activate muscle quickly are likely candidates. In contrast, fatiguing MVICs with a rapid intent appear to increase corticospinal inhibition, but more research is needed to verify this finding and identify if the inhibition is of intracortical or spinal origin.

## Data Availability

The raw data supporting the conclusions of this article will be made available by the authors, without undue reservation.
